# Body surface scan anthropometrics are related to cardiorespiratory fitness in the general population

**DOI:** 10.1038/s41598-022-26740-8

**Published:** 2022-12-23

**Authors:** Armin Köhler, Berit Filges, Henry Völzke, Stephan B. Felix, Ralf Ewert, Beate Stubbe, Marcello R. P. Markus, Stefan Groß, Marcus Dörr, Till Ittermann, Martin Bahls

**Affiliations:** 1 Institute for Internal Medicine B, University Medicine Greifswald, Ferdinand-Sauerbruch-Straße, 17475 Greifswald, Germany; 2 Institute for Community Medicine, Department SHIP - Clinical-Epidemiological Research, University Medicine Greifswald, Greifswald, Germany; 3 German Centre for Cardiovascular Research (DZHK) Partner Site Greifswald, Greifswald, Germany

**Keywords:** Diagnostic markers, Predictive markers, Diagnosis, Public health, Epidemiology

## Abstract

The assessment of cardiorespiratory fitness (CRF) is an important tool for prognosis evaluation of cardiovascular events. The gold standard to measure CRF is cardiopulmonary exercise testing (CPET) to determine peak oxygen uptake (VO2peak). However, CPET is not only time consuming but also expensive and is therefore not widely applicable in daily practice. The aim of our study was to analyze, whether and which anthropometric markers derived from a 3D body scanner were related to VO2peak in a general population-based study. We analyzed data (SHIP-START-3) from 3D body scanner and CPET of 1035 subjects (529 women; 51.1%, age range 36–93). A total of 164 anthropometric markers were detected with the 3D body scanner *VITUS Smart XXL* using the software *AnthroScan Professional*. Anthropometric measurements were standardized and associated with CRF by sex-stratified linear regression models adjusted for age and height. Anthropometric markers were ranked according to the  − log- *p* values derived from these regression models. In men a greater left and right thigh-knee-ratio, a longer forearm-fingertip length, a greater left thigh circumference and greater left upper arm circumference were most strongly associated with a higher VO2peak. In women a greater left and right thigh circumference, left calf circumference, thigh thickness and right calf circumference were most strongly associated with a higher VO2peak. The detected VO2peak-related anthropometric markers could be helpful in assessing CRF in clinical routine. Commonly used anthropometric markers, e.g. waist and hip circumference, were not among the markers associated with VO2peak.

## Introduction

Cardiovascular diseases (CVD) are responsible for one-third of all deaths in the adult population^1^. The burden of CVD might be reduced by early risk detection and preventive strategies^2^. The assessment of cardiorespiratory fitness (CRF) is an important tool for prognosis of cardiovascular events^3^. Low CRF is an independent risk factor for CVD as well as cardiovascular and all-cause mortality in the general population^4–6^. CRF decreases with age and that decrease is further accelerated by unhealthy and sedentary lifestyles that are highly prevalent in Western societies^7^. The gold standard to measure CRF is cardiopulmonary exercise testing (CPET) to determine the peak oxygen uptake (VO2peak) as a central parameter. However, CPET is not only time consuming but also expensive and is, therefore, not widely applicable in daily practice. For clinical assessment it would be helpful to identify predictors of CRF that are easier to obtain such as anthropometric parameters. Waist circumference (WC) is a widely used example of an anthropometric marker^8^. Previous studies found a low CRF in individuals with high WC^9–11^.

Additively a sport specific study found strong correlations between calf girth, hip girth, height, sitting height, limb length and shoulder breadth with VO2peak for rowers, which may be based on better leverages. Moreover various studies did point out waist-to-height ratio as a better screening tool than WC and body mass index (BMI) for adult cardiometabolic risk factors^12–14^. In line with these findings is a study in 1077 Japanese women which suggested that the waist-to-height ratio may be a better predictor of multiple Coronary Heart Disease (CHD) than BMI or the waist-to-hip ratio (WHR)^15^.

Beyond WC and WHR there may be other body surface related anthropometric parameters, which may be even more tightly related to CRF. The advantage of anthropometric parameters is that the assessment of them is cheaper and takes less time compared to CPET. In clinical practice symptom related anthropometric markers could help to estimate CRF and optimize therapy.

The aim of our study was to analyze associations of 164 anthropometric markers derived from a 3D body scanner with CRF as determined by VO2peak from a CPET in a population-based study. With this systematic approach we aimed to identify anthropometric markers, which are highly predictive for VO2peak and, thus, may add value for the clinical setting. This study may be used as a first step in identifying new anthropometric indicators that may be measured in clinical settings using cheaper tools such as measuring tape.

## Methods

### Study design and participants

The Study of Health in Pomerania (SHIP) is a population-based study assessing a multitude of risk factors, resources and determinants of health and disease in the adult population of West Pomerania, a region in the northeast of Germany^16,17^. A sample of the 212,157 inhabitants was selected from population registrar’s office. A representative sample, comprising 7008 adults aged 20–79 years with 292 persons of each sex in each of the twelve 5 year age strata, was drawn. The net sample (without migrated or deceased people) consisted of 6267 eligible subjects, of whom 4308 individuals participated in the baseline study of SHIP-START-0 from 1997 to 2001.

**Figure 1:**
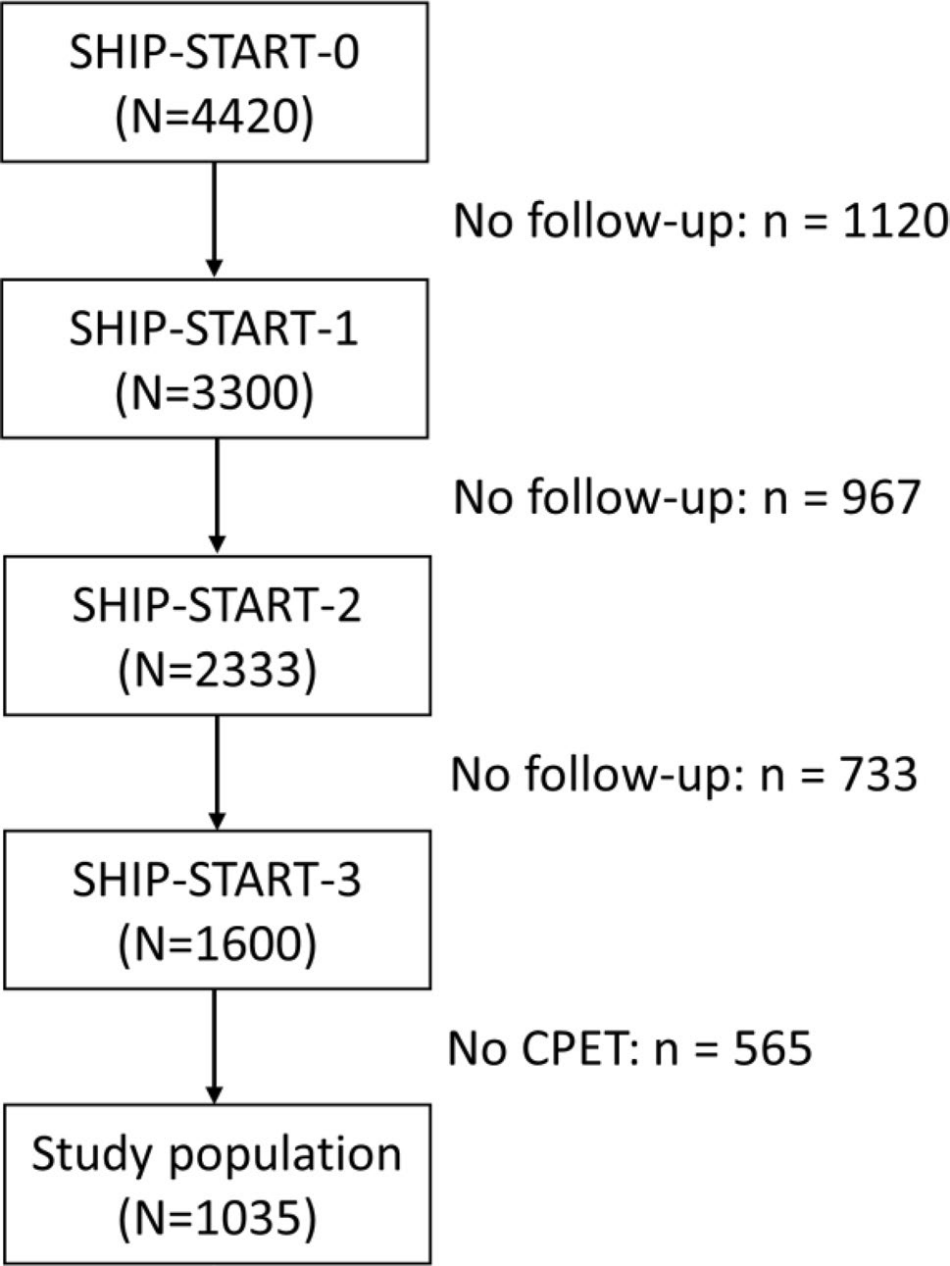
PRISM diagram about the selection of participants.

We used data from the 3rd follow-up, SHIP-START-3, which was conducted between February 2014 and March 2016. Data from 3D body scanner and CPET was available in 1,035 participants (529 women; 51.1%, age range 36–93). A PRISM diagram shows the selection of participants (Fig. 1). A detailed description of measurement and analysis of the 3D body scanner data can be found in the supplement.

The study complies with the Declaration of Helsinki and was approved by the ethics committee of the University of Greifswald. All study participants gave written informed consent.

### Interview, medical and laboratory examination

Information on age, sex, physical activity (PA), smoking status (never, former or current smoker), medication use and medical history was gathered by trained and certificated staff during a standardized computer-assisted interview. Individuals who did not participate in leisure time physical activity (LTPA), for at least one hour per week, during summer or winter were classified as sedentary^18^. With the Baecke score we measured habitual sport related PA (SPA), work related PA (WPA) and leisure time related PA (LTPA)^19,20^. Domain specific physical activity was assessed using the well-established Baecke questionnaire^19,20^. Briefly, the questionnaire consists of 16 questions in three distinct sections: physical activity during leisure time excluding sport (i.e. one’s own LTPA compared to others of similar age as well as sweating, playing sports, watching television, walking and cycling during leisure time), SPA (i.e. identification of the sports played followed by questions regarding duration per week and months per year) and WPA (i.e. one’s own WPA compared to others of similar age followed by questions regarding sitting, standing, walking, lifting of heavy loads and sweating at work as well as if one is tired after work). Most questions are scored on a five-point Likert scale, ranging from never to always or very often. For the reported sport activities, additional questions query the number of months per year and hours per week of participation. The three derived indices, LTPA, SPA and WPA, are scored in arbitrary units ranging from 1 to 5.

Information on medication intake was categorized according to the anatomical therapeutic chemical classification (ATC) code^16,21^. Standardized measurement of weight was performed with calibrated scales. Body mass index (BMI) was calculated as weight (kg)/height^2^ (m^2^). After a resting period of at least five minutes systolic and diastolic blood pressure were measured three times on the right arm of seated subjects using an oscillometric digital blood pressure monitor (HEM-705CP, Omron Corporation, Tokyo, Japan) with an interval of three minutes between readings. For the present analyses the mean of the second and third measurements was used. Hypertension was defined as a systolic blood pressure ≥ 140 and/or a diastolic blood pressure ≥ 90 mmHg or use of antihypertensive medication (ATC codes C02, C03, C04, C07, C08 and C09).

Non-fasting venous blood samples were obtained from all study participants between 07:00 a.m. and 04:00 p.m. while sitting^21^. Serum aliquots were stored at − 80 °C. Glycosylated hemoglobin was determined by high-performance liquid chromatography (Diamat, Bio-Rad Laboratories, Munich, Germany). Glucose concentration was measured photometrically (Dimension RxL or Dimension VISTA analyzers, Siemens Healthcare Diagnostics, Eschborn, Germany). Diabetes mellitus was defined as self-reported and/or use of antidiabetic medication (ATC code A10) and/or glycated hemoglobin ≥ 6.5% and/or fasting glucose ≥ 7.0 mmol/l and/or non-fasting glucose ≥ 11.1 mmol/l. History of myocardial infarction was self-reported.

### Body surface scan

**Figure 2:**
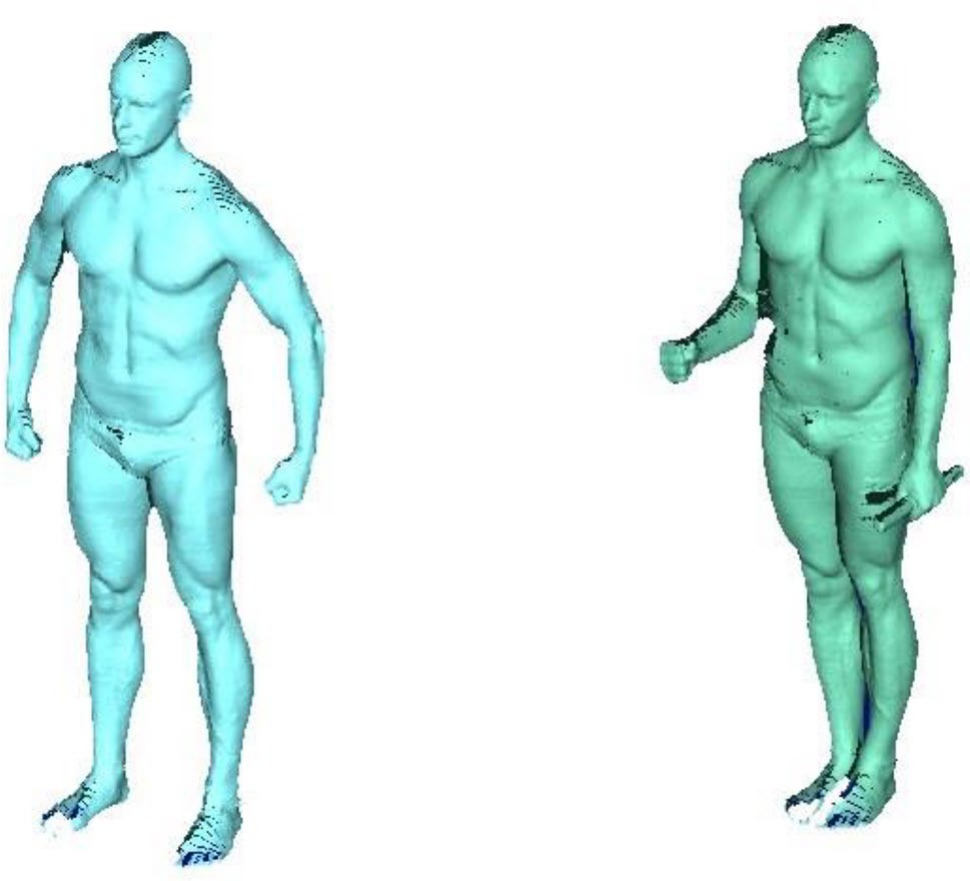
Typical standing upright positions with fixed points based on manufacturer’s instructions. Typical standing upright position without (left image) and with wooden dipstick (right image). On right image the left hand holds a small wooden stick (“dipstick”) vertically. The images were provided with kind permission of the author.

Previous studies verified the validity of anthropometric measurements using a 3D body scanner^22,23^. Anthropometric markers were measured with a 3D body scanner (VITUS Smart XXL, Vitronic, Wiesbaden, Germany) using the software AnthroScan Professional (Version 3.0.7, Human Solutions GmbH, Kaiserslautern, Germany) which provides a 3D image of the body surface. Based on four eye-safe lasers, eight cameras, and optical triangulation, the body surface scan generates a 3D point cloud depicting the body surface within 12 s^8^. Following international norms^24^, 164 anthropometric measures were determined from the 3D picture (accuracy, ± 1 mm; density, 27 points/cm^2^; around 500,000 points/scan), e.g. WC, arm circumference, etc. The body surface scan was calibrated daily using a cylinder tube of defined measurements according to the manufacturer’s instructions. All tests were performed at room temperature, because the measurement was performed in ideally tight-fitting underwear wearing a bathing cap to cover hair. Each participant was instructed to stand upright with head positioned according to the Frankfort Horizontal plane^25,26^, legs hip wide positioned, arms slightly angled without body contact, and hands making a fist with thumbs outside showing forward, if possible. Breathing should be normal. (Fig. 2) After each scan, 3D pictures were quality checked by study personnel, i.e., for image errors or artefacts, or deviations from standard posture.

We followed the guidelines of the International Organization for Standardization (ISO-7250) to measure the anthropometric markers^27^. The room was darkened and the measuring chamber was closed except for a small gap. This allows the study nurse to supervise the scan posture of the participant^28^. In a prepared scan wizard, the body scan according to ISO guidelines was started in a sitting position^29^. More scanning details and a detailed description of the standard scanning procedure can be found in supplement.

### Exercise testing and gas exchange variables

A symptom-limited exercise test using a calibrated electromagnetically braked cycle ergometer (*Ergoselect 100*, Ergoline, Germany) was performed according to a modified Jones protocol (stepwise increase in work load of 16 Watts/minute, starting with unloaded cycling plus the ergometer related permanent load) as previously described^30–32^. All tests were performed according to current guidelines for exercise testing^33^, with continuous monitoring of ECG, blood pressure and oxygen saturation. Gas exchange and ventilatory variables were analyzed breath by breath averaged over 10 s intervals using a computer-based system. Exercise duration was measured from the start of exercise (without resting period) up to its termination. In the absence of chest pain and ECG abnormalities, all tests were continued as symptom-limited (volitional exertion, dyspnea or fatigue). VO2peak was defined as the highest 10 s average of absolute oxygen uptake in the last minute of exercise^32^. A sex-specific analysis was performed a priori due to sex-specific differences of anthropometric markers for VO2peak^34^. The median time interval between body scan examination and CPET was 21 days (inter-quartile range, IQR, 6; 49 days).

### Statistical analysis

**Figure 3:**
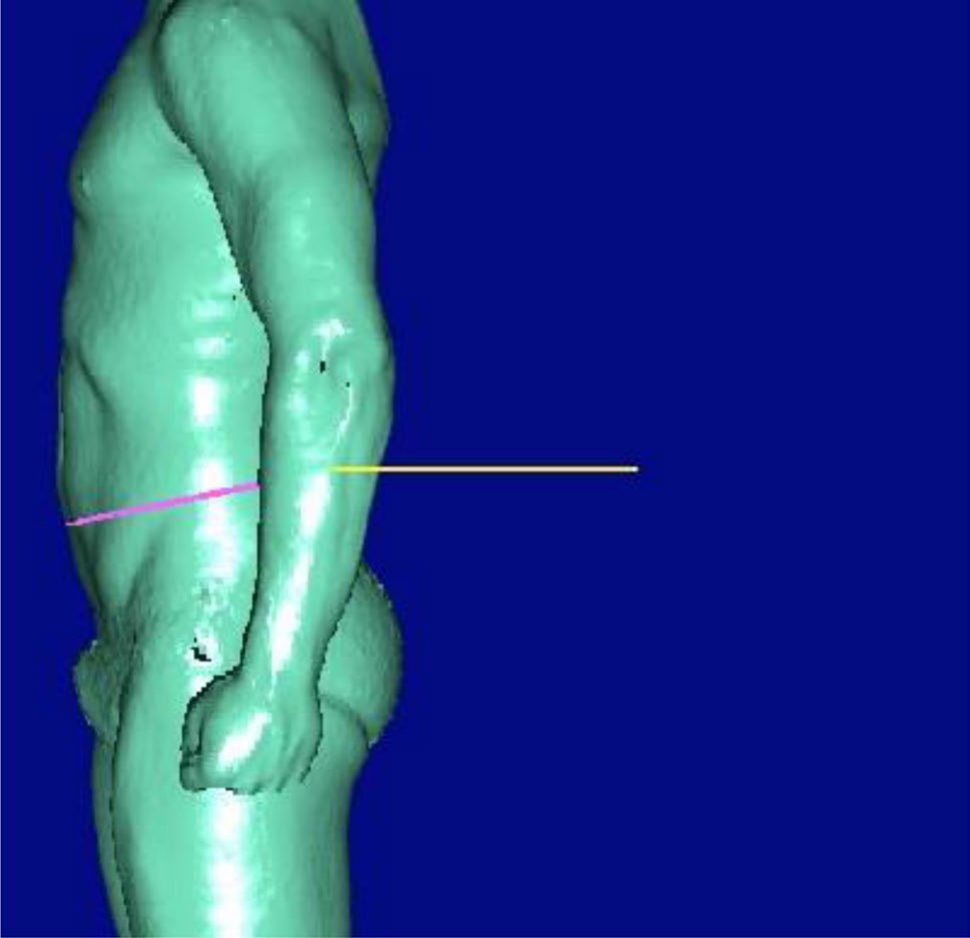
“Distance back to the wall” as unsuitable parameter for daily practice. This image was provided with kind permission of the author.

From the 164 markers derived from body scanning we excluded parameters which were highly correlated with variables already selected, e.g. shoulder height measured in sitting position was not used because sitting height was already selected or shoulder breadth and acromial breadth. Moreover, we deleted parameters which one cannot measure in clinical practice e.g. distance back to the wall (Fig. 3). The resulting 87 variables (see supplement for full list) were standardized and used separately in sex-stratified linear regression models adjusted for age and height with VO2peak as outcome. As a result we derived for each variable a β-coefficient, a 95%-confidence interval (CI) and a -log p value. For each sex the β-coefficients and 95%-confidence intervals for the twenty variables with the highest  − log *p* values were plotted. The β-coefficients, 95%-confidence intervals and − log *p* values for all of the selected 87 parameters are provided in the supplementary material. To account for multiple testing, we applied a Bonferroni correction. A *p* value ≤ 0.000287 (− log(*p*) ≥ 3.542) was considered as statistically significant. All statistical analyses were performed using Stata 16.0 (Stata Corporation, College Station, TX, USA).

## Results

### Characteristics of the study sample

The main characteristics of the study population are provided in Table 1. Men and women had a median age of 59.3 years (IQR, 36.0 to 93.0 years). Men had a BMI of 28.2 kg/m^2^. Women, on the other hand, had a BMI of 27.1 kg/m^2^. HbA1c was 5.8% in men and 5.7% in women. The absolute VO2peak was 2281.2 ml/min for men and 1610.6 ml/min in women.

**Table 1:** Descriptive statistics of the study population.

	Men	Women	Total
Parameter	Mean (SD)	Mean (SD)	Mean (SD)
Age	60.3 (13.1)	58.4 (11.6)	59.3 (12.4)
Waist circumference (cm)	101.4 (11.4)	89.3 (13.2)	95.2 (13.8)
Hip circumference (cm)	101.2 (8.0)	103.1 (11.3)	102.2 (9.9)
Alcohol (g/day)	16.7 (19.2)	6.5 (7.8)	11.5 (15.4)
Body mass index (kg/m^2^)	28.2 (3.9)	27.1 (5.0)	27.6 (4.5)
Weight (kg)	87.1 (13.8)	72.4 (13.9)	79.6 (15.7)
Height (cm)	175.7 (6.6)	163.4 (6.6)	169.4 (9.0)
Systolic BP (mmHg)	134.4 (14.4)	127.9 (15.1)	131.1 (15.1)
Diastolic BP (mmHg)	78.6 (10.2)	76.5 (8.7)	77.5 (9.5)
Sports index	2.6 (0.7)	2.7 (0.6)	2.7 (0.7)
Leisure time index	3.2 (0.6)	3.2 (0.6)	3.2 (0.6)
HbA1c (% Hb)	5.8 (0.6)	5.7 (0.6)	5.7 (0.6)
Triglycerized (mmol/l)	2.0 (1.4)	1.5 (1.0)	1.7 (1.2)
Total cholesterol (mmol/L)	5.2 (1.1)	5.5 (1.1)	5.4 (1.1)
LDL-C (mmol/L)	3.3 (0.9)	3.4 (0.9)	3.3 (0.9)
HDL-C (mmol/L)	1.3 (0.3)	1.6 (0.4)	1.5 (0.4)
Vo2peak (ml/min)	2281.2 (647.4)	1610.6 (366.2)	1938.5 (621.0)
Vo2peak/kg (ml/min/kg)	27.5 (7.5)	23.4 (5.5)	25.4 (6.9)
**Smoking status n (%)**			
Never	144 (28.5)	252 (47.6)	396 (38.3)
Former	287 (56.7)	190 (35.9)	477 (46.1)
Current	75 (14.8)	87 (16.5)	162 (15.7)
Diabetes mellitus n (%)	63 (12.6)	53 (10.1)	116 (11.3)
Myocardial infarction n (%)	18 (3.6)	7 (1.3)	25 (2.4)
Antihypertensive medications n (%)	226 (44.7)	200 (37.7)	426 (41.1)

*HbA1c* glycated hemoglobin, *LDL-C* low density lipoprotein cholesterol, *HDL-C* high density lipoprotein cholesterol, *VO2peak* peak oxygen uptake, *VO2peak/kg* body weight adjusted peak oxygen uptake, *BP* blood pressure.

### Associations between anthropometric markers and VO2peak

**Figure 4:**
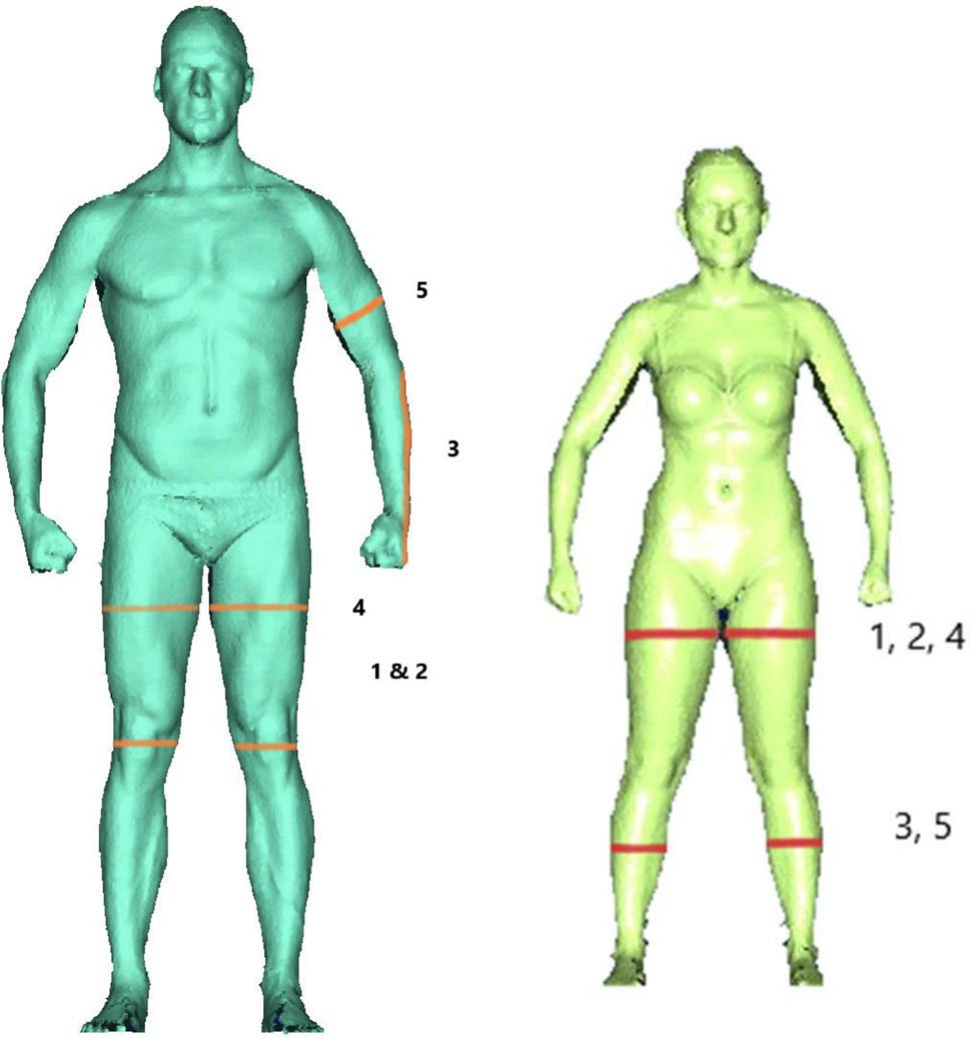
The five strongest related body scan markers for VO2peak (ml/min) for male and female subjects; strongest associations for men are colored in orange and for women in red. The images were provided with kind permission of the authors.

In men the five strongest associations of anthropometric markers with a higher VO2peak were found for a greater left thigh-knee-ratio (β − 146.5; − log *p* 11.3; CI (− 208.4 to − 84.6), respectively) and a greater right thigh-knee-ratio (β − 129.8; − log *p* 9.9; CI (− 190.3 to − 69.3)), a longer forearm-fingertip length (β 102.5; − log *p* 5.3; CI (30.9 to 173.9)), a greater left thigh circumference (β 96.8; − log p 7.3; CI (41.4 to 152.3)) and a greater left upper arm circumference (β 93.0; − log *p* 7.7; CI (41.4 to 144.6)). (Table 2 and Fig. 4).

**Table 2:** Characteristics of the strongest anthropometric markers related to VO2peak for men.

Variable	Rank	Beta (95% CI)	*p* value	R^2^
Left thigh knee ratio	1	140 (77.84, 201.82)	12.32	0.39
Right thigh knee ratio	2	126 (65.9, 186.16)	10.40	0.38
Left upper arm circumference	3	93 (41.42, 8144.58)	7.71	0.38
Right thigh circumference	4	93 (39.89, 145.89)	7.35	0.38
Left thigh circumference	5	97 (41,36, 152,26)	7.31	0.38
Right upper arm circumference	6	89 (37.14, 141.46)	7.07	0.37
Right calf circumference	7	75 (25.00, 124.68)	5.69	0.37
Left forearm circumference	8	76 (24.82, 127.74)	5.56	0.37
Forearm-fingertip length	9	102 (30.99, 173.99)	5.27	0.37
Right forearm circumference	10	72 (21.40, 123.22)	5.19	0.37
Left calf circumference	11	64 (14.24, 113.24)	4.43	0.37
Left upper elbow ratio	12	60 (13.19, 106.83)	4.07	0.37
Thigh thickness	13	62 (11.16, 113.72)	4.05	0.37
Left upper arm diameter	14	60 (8.52, 110.48)	3.97	0.37
Right upper elbow ratio	15	61 (8.52, 112.84)	3.79	0.36
Length waist buttock	16	73 (8.03, 138.03)	3.57	0.37
Right elbow circumference	17	53 (5.59, 101.27)	3.57	0.37

Detailed explanation of the parameters see supplementary Table 1.

**Figure 5:**
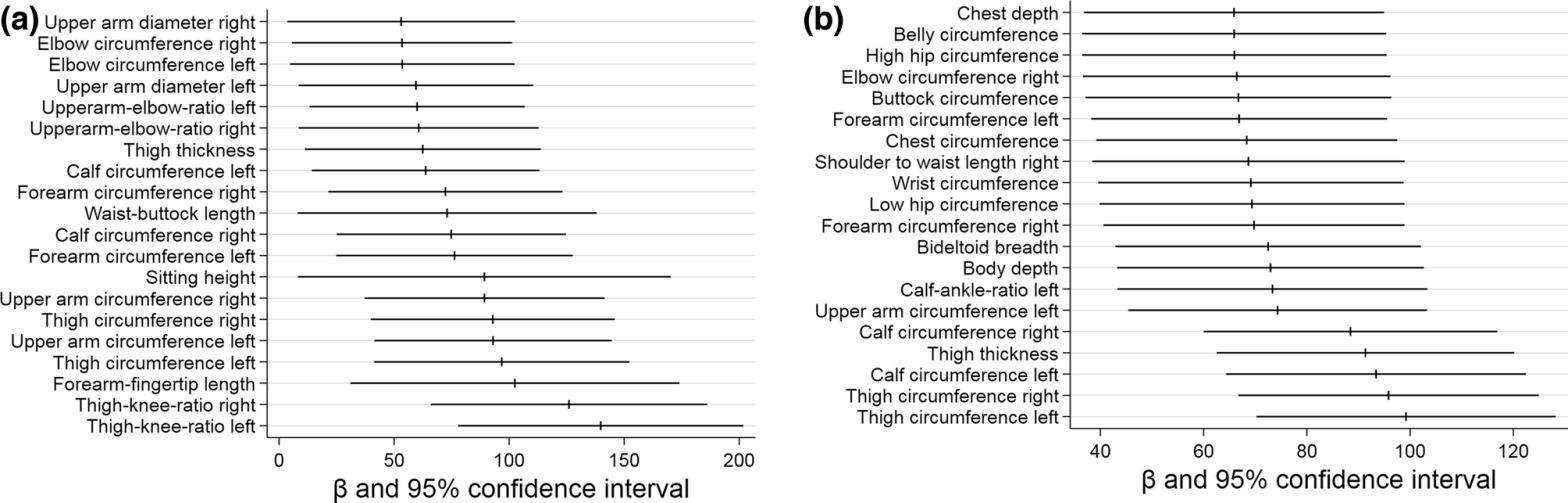
(**a**) Graphics of the strongest related body scan markers for VO2peak (ml/min) for male subjects; the strongest associations are displayed in the lower right; lines are expressed as β- and 95% confidence interval. (**b**) Graphics of the strongest related body scan markers for VO2peak (ml/min) for female subjects (graph below); strongest associations are displayed in the lower right; lines are expressed as β- and 95% confidence interval.

In women, the five strongest higher VO2peak-related anthropometric markers were a greater left thigh circumference (β 99.2; − log *p* 23.7; CI (70.2 to 128.2)) and a greater right thigh circumference (β 95.8; − log *p* 22.1; CI (66.7 to 124.9)), a greater left calf circumference (β 93.4; − log *p* 21.2; CI (64.4 to 122.5)), a greater thigh thickness (β 91.4, − log p 20.7; CI (62.5 to 120.2)) and a greater right calf circumference (β 88.5; − log p 19.9; CI (59.9 to 116.9)) (Table 3 and Fig. 4). For a better overview Figs. 5a,b display significant anthropometric parameters derived by body scan that showed the highest associations with VO2peak adjusted for age and height (all − log(*p*) ≥ 3.542; Fig. 5a men, Fig. 5b women).

**Table 3:** Characteristics of the strongest anthropometric markers related to VO2peak for women.

Variable	Rank	Beta (95% CI)	*p* value	R^2^
Left thigh circumference	1	99 (70.22, 128.20)	23.69	0.32
Right thigh circumference	2	96 (66.70, 124.94)	22.08	0.32
Left calf circumference	3	93 (64.35, 122.45)	21.19	0.3
Thigh clearance	4	91 (62.54, 120.16)	20.67	0.3
Right calf circumference	5	88 (59.99, 116.91)	19.95	0.3
Left upper arm circumference	6	74 (45.39, 103.27)	14.23	0.28
Body depth	7	73 (43.24, 102.68)	13.51	0.28
Bideltoid breadth	8	72 (42.89, 102.09)	13.14	0.28
Left calf ankle ratio	9	73 (43.34, 103.36)	13.08	0.28
Right forearm circumference	10	70 (40.61, 98.91)	12.56	0.28
Hip circumference	11	69 (39.82, 98.90)	12.16	0.28
Bust circumference	12	68 (39.19, 97.51)	12.12	0.28
Wrist circumference	13	69 (39.59, 98.73)	12.07	0.28
Left forearm circumference	14	67 (38.23, 95.53)	12.03	0.27
Length neck right bust waist	15	69 (38.41, 98.93)	11.45	0.28
Chest depth	16	66 (36.79, 94.97)	11.41	0.28
Buttock circumference	17	67 (37.11, 96.37)	11.30	0.28
Belly circumference	18	66 (36.45, 95.37)	11.18	0.28
High hip circumference	19	66 (36.45, 95.47)	11.16	0.28
Right elbow circumference	20	66 (36.62, 96.22)	11.10	0.28

## Discussion

We analyzed the associations between multiple anthropometric markers derived from a 3D body scanner and objectively measured VO2peak based on data from a large population-based study. We showed that thigh-knee-ratios, forearm-fingertip length, left thigh circumference and left upper arm circumference were the anthropometric markers most strongly associated with VO2peak in men. In women, thigh circumference, calf circumferences and thigh thickness were associated with VO2peak. Many of the identified anthropometric markers related with VO2peak were located in the lower extremities, commonly used torso based measurements like WC, hip circumference (HC) and WHR, were not significantly associated with VO2peak. Our findings potentially support the use of anthropometric markers as predictors for CRF if there is no access or no time for CPET. Their precision in estimating CRF could improve clinical assessment of patients.

### Modifiable anthropometric markers

We previously reported that an inverse association between CRF and mortality^20^. Historically, anthropometric markers have been used to characterize physical health. For example, mid-upper arm muscle circumference has been used as a marker of protein-energy malnutrition^35,36^. Hence, this measurement may provide a direct assessment of underlying muscle which is representative of skeletal muscle mass. Skeletal muscle mass may be increased by strength training. In men the strongest association for VO2peak were thigh circumference values. Greater thigh-knee-ratios were strongly associated with a higher VO2peak in men. A large thigh-knee-ratio is defined by thin knees and thick thighs. In order to influence this ratio one could increase thigh thickness through appropriate training or decrease knee thickness by lowering fat tissue around the knee. Moreover, an average healthy, untrained person, should get more muscular thighs through adequate training^37^.

Previous studies showed inverse associations between CRF and WC^38,39^. High CRF was associated with a lower BMI, independent of gender^40^. Here we report that other anthropometric markers besides the WC show stronger associations with CRF. This could be related to the fact that previous studies simply did not have a 3D body scanner available to test other anthropometric markers. Therefore, “new” leg circumference based markers may be taken into account. Despite the fact that HC and WC were not significantly associated with VO2peak in our study population, we acknowledge that these measures are still clinically meaningful due to their association with morbidity and mortality^10^.

### Fixed anthropometric markers

In contrast to modifiable anthropometric markers, fixed anthropometric markers such as upper arm length or leg length are genetically determined. The values of fixed anthropometric variables change very little or not at all upon full physical body development^41^. The forearm-fingertip length, which showed a strong association with VO2peak in men, is mainly based on the sum of bone length of the forearm and hand, which are mostly genetically determined and/or childhood influenced. While one may argue that this could be a finding by chance, a previous study reported that the right-left digit ratio (2D:4D) was also related to maximal oxygen uptake. The authors could show that a low 2D:4D ratio is associated with sensitivity to testosterone measured by the number of cytosine-adenine-guanine triplet repeats in exon 1 of the androgen receptor gene^42^. Another parameter of a strong association with the VO2peak is sitting height in men but not in women.

Moreover, the length of extremities in certain competitive sports can be seen as performance-determining part. Athletes with short height and extremities, e.g. arm and leg length, are usually not found in world class competitions in high jump, sprint, basketball, handball^43^, volleyball, rowing^44^ and swimming^45^. A study among a group of fifteen male elite and fifteen university rowers found strong relationships between manually measured anthropometric markers (i.e., calf and hip girth, height, length of extremities and shoulder breadth) to fitness level variables in an ex-post-facto design. Therefore, one may assume that anthropometrics and fitness level have a strong relationship toward rowers’ performance^44^.

This could point to a new estimation approach of VO2peak indicating fixed anthropometric parameters. Furthermore, it must be taken into account that a certain part of CRF is also genetically determined^46^. Conclusively, fixed anthropometric markers could be used to indicate someone’s baseline level of CRF.

### Study limitations and strengths

Even though, SHIP comprises a population-based and methodologically rigorous regionally representative survey of Germany, the findings of our analyses need to be interpreted in the context of several limitations. First, our analysis consisted of white Europeans living in rural areas and it is not known whether our results are also applicable to other ethnicities^20^. Second, in order to confirm which anthropometric marker is the most appropriate, the ability of each of these anthropometric markers to predict certain events needs to be analyzed in further studies. These strong associations of anthropometric markers with a higher VO2peak should be used as indicators in clinical practice. To predict certain events the physician needs to take various clinical markers into account and weighing them up wisely for his general view of the patient. Our findings may help the physician in making diagnosis.

Irrespective of these limitations, strengths of our study are the population-based setting, the use of standardized data collection methods, the capacity to perform adjustment for a variety of clinical risk factors and the availability of CPET results from 1035 participants.

## Conclusion and future direction

The detected VO2peak-related anthropometric markers in this study could be helpful in estimating CRF in clinical routine. Commonly used anthropometric markers, e.g. WC and HC, did not show the strongest association with VO2peak. Potential interventions to increase VO2peak may measure modifiable anthropometric markers like thigh-knee-ratios in men or thigh circumferences in women. Fixed anthropometric markers should be investigated in the future to assess whether these are findings by chance or if they are indeed biologically related to CRF. In conclusion, future research should assess whether the sex-specific anthropometric markers identified in this study can be used to improve CVD risk assessment in the general population.

## Supplementary Information

Supplementary Information.

## Data Availability

Data from the “Study of Health of Pomerania” are available from the University Medicine Greifswald, Germany but restrictions apply to the availability of these data, which were used under license for the current study, and so are not publicly available. Data are, however, available upon reasonable request at https://www.fvcm.med.uni-greifswald.de/dd_service/data_use_intro.php and with permission of the University Medicine Greifswald.
