# Supplementary material for: Body surface scan anthropometrics are related to cardiorespiratory fitness in the general population
Source: Sci Rep. 2022 Dec 23;12:22185. doi: 10.1038/s41598-022-26740-8 (PMC9789061; doi:10.1038/s41598-022-26740-8)
Supplement: Supplementary file 1 — Supplementary Information. [file 41598_2022_26740_MOESM1_ESM.docx]

**Supplement**

1. **Procedure of clinical measurements / body surface scan**
2. **Anthropometry**
3. **Supplementary table 1: Description of anthropometric markers**
4. **Supplementary table 2: Ranking of -log p values: 164 markers of men and women**
5. **Supplementary table 3: Correlation coefficients between the anthropometric markers**

**1 Procedure of clinical measurements / body scan**

This posture is an upright posture for determining ISO-7250 measurements in standing position as well as height.

The participant was asked to do the following:

- stand on the measuring platform
- to stand with the feet closed
- to stand upright ("spread out")
- to let the left arm hang down freely, the left hand holds the measuring device at the sagittal plane with horizontal grip axis - to let with the right upper arm hang down relaxed
- to hold the right forearm horizontally forward at right angle, to hold the hand as a relaxed fist
- to keep the head in an horizontally position and looking forward (Frankfort Horizontal)^18^
- to continue breathing normally

This allows the examiner to supervise the scan posture of the patient. In a prepared scan wizard, the ISO scan is started in a standing position.

**Standard scanning procedure**

This position is the relaxed basic posture for determining general body measurements such as height, length and girth measurements.

The participant was asked:

- to stand on the measuring platform
- to place the feet on the foot markers and, depending on the width of the shoulders, to reduce or increase the distance between the feet (instructions given by the examiner)
- to stand upright
- to clench his hands to his fist and keep them parallel to the body at the level of the trouser seam
- to stretch the arms slightly away from the body and bend them (instructions: carrying heavy suitcases)
- to keep the head horizontal and facing forward (Frankfort Horizontal)^18^
- to continue breathing normally

The room was darkened and the measuring chamber was closed except for a gap. This allowed the examiner to supervise the scan posture of the test person. In the prepared Scan-Wizard the standard scan was started.

We followed the guidelines of the International Organization for Standardization (ISO-7250) to measure the anthropometric markers:^20^

- to stand or sit in an upright posture on the scan chair
- in sitting position: the chair was adjusted to the body size, knee angle 90° in sitting position with ground-touching feet
- in sitting position: thighs are completely resting on the chair
- in sitting position: to sit up straight ("spread out") - allow upper arms to hang down freely
- in sitting position: to hold the right forearm at a right angle horizontally forward, the right hand holds a small wooden stick (“dipstick”) vertically
- in sitting position: to hold the left forearm at a right angle horizontally forward, stretch out the left hand with closed fingers
- in standing position: to hold the right forearm at a right angle horizontally forward, the left hand holds a small wooden stick (“dipstick”) vertically
- in standing position without wooden stick: to hold the upper arms slightly stretched from torso, allow forearms to hang down freely
- to hold the head horizontally and looking forward (Frankfort Horizontal)^18^

to continue breathing normally

1. **Anthropometry**

After the first two scans, the patient was asked to leave the scanner to perform the manual measurements of somatometry (for details, see supplement: SOP Somatometry).

During the somatometric measurements, five physical markers (Figure 3) are attached to the front left side of the subject's body. It is important to place the markers in correct height; a lateral offset is unproblematic and, in many cases, necessary: To clearly identify the markers in the scan, two markers must be at least 4 cm apart from each other.  To avoid also problems with the shadow of the arms (especially with obese persons), the markers cannot be attached directly to the side of the test person, but to the left front side of the body.

Three anatomical points are palpated and marked:

- last rib

- iliac crest

- points of the large rolling hills (thigh bone, large trochanter)

that protrude furthest laterally

The palpation is carried out on the side of the body in extension of the trouser seam, the markers are attached at the corresponding body height (horizontal offset possible, see above).

In addition, two parameters at the positions of the manual somatometric measurements

(see **figure 3**):

- Waist circumference measurement
- Hip circumference measurement

Measurement and data are in accordance with ISO 20685 the standard for 3D scanning methodologies for internationally compatible anthropometric databases.

The scanner was calibrated daily before data collection using a standard calibration body. Body measures were extracted automatically by the scanner software using default parameter settings as implemented by the manufacturer which includes reference point identification and gap-filling algorithms.

1. **Supplementary table 1:** **Description of anthropometric markers**

| **Anthropometric marker** | **Description** |
| --- | --- |
| belly circumference | belly circumference; standing position (cm) |
| bideltoid breadth | shoulder breadth measured between musculi deltoidei; horizontal distance (cm) |
| body depth | body depth; standing position (cm) |
| buttock circumference | buttock circumference; standing position (cm) |
| calf-ankle-ratio left | circumference calf left (cm)/circumference ankle left (cm); standing position |
| calf-ankle-ratio right | circumference calf right (cm)/circumference ankle right (cm); standing position |
| calf circumference left | calf circumference left; standing position (cm) |
| calf circumference right | calf circumference right; standing position (cm) |
| chest circumference | chest circumference; standing position (cm) |
| chest depth | horizontal chest depth; standing position (cm) |
| elbow circumference left | elbow circumference left; standing position (cm) |
| elbow circumference right | elbow circumference right; standing position (cm) |
| forearm circumference left | forearm circumference left; standing position (cm) |
| forearm circumference right | forearm circumference right; standing position (cm) |
| forearm-fingertip length | forearm-fingertip length (cm) |
| high hip circumference | higher hip circumference; standing position (cm) |
| low hip circumference | lower hip circumference; standing position (cm) |
| right shoulder-right chest-waist-length | length from the right shoulder over the right chest to the waist circumference; standing position; measurement along the surface (cm) |
| sitting height | sitting height in an upright position; vertical distance (cm) |
| thigh circumference left | thigh circumference left; horizontal measurement; standing position (cm) |
| thigh circumference right | thigh circumference right; horizontal measurement; standing position (cm) |
| thigh-knee -ratio left | circumference thigh left (cm)/circumference knee left (cm); standing position |
| thigh-knee-ratio right | circumference thigh right (cm)/circumference knee right (cm); standing position |
| thigh thickness | thigh thickness; vertical distance; sitting position (cm) |
| upper arm circumference left | upper arm circumference left; standing position (cm) |
| upper arm circumference right | upper arm circumference right; standing position (cm) |
| upper arm diameter left | upper arm diameter left; horizontal measurement; standing position (cm) |
| upperarm-elbow-ratio left | circumference upperarm left (cm)/circumference elbow left (cm); standing position |
| upperarm-elbow-ratio right | circumference upperarm right (cm)/circumference elbow right (cm); standing position |
| waist-buttock length | dorsal length from waist circumference to buttock circumference; standing position; measurement along the surface (cm) |
| wrist circumference | wrist circumference; standing position (cm) |

1. **Supplementary table 2: Ranking of -log p values: 164 parameters of men and women**

For a better estimation of the effect of the body scan marker the R² for basic models (including age and height) **without body scan marker** was calculated for **men** **R² = 0.3671** and **women** **R² = 0.2536**. The ranking remains the same.

| **Variable** | **Gender** | **Rank** | **Beta** | **lb** | **ub** | **pvalue** | **r2** |
| --- | --- | --- | --- | --- | --- | --- | --- |
| left thigh circ | Females | 1 | 99 | 70,22 | 128,20 | 23,6883 | 0,32 |
| right thigh circ | Females | 2 | 96 | 66,70 | 124,94 | 22,0804 | 0,32 |
| left calf circ | Females | 3 | 93 | 64,35 | 122,45 | 21,1933 | 0,30 |
| thigh clearance | Females | 4 | 91 | 62,54 | 120,16 | 20,6745 | 0,30 |
| right calf circ | Females | 5 | 88 | 59,99 | 116,91 | 19,9493 | 0,30 |
| left upperarm circ | Females | 6 | 74 | 45,39 | 103,27 | 14,2269 | 0,28 |
| body depth | Females | 7 | 73 | 43,24 | 102,68 | 13,5142 | 0,28 |
| bideltoid breadth | Females | 8 | 72 | 42,89 | 102,09 | 13,1386 | 0,28 |
| left calf ankle ratio | Females | 9 | 73 | 43,34 | 103,36 | 13,0844 | 0,28 |
| right forearm circ | Females | 10 | 70 | 40,61 | 98,91 | 12,5629 | 0,28 |
| hip circ | Females | 11 | 69 | 39,82 | 98,90 | 12,1570 | 0,28 |
| bust circ | Females | 12 | 68 | 39,19 | 97,51 | 12,1205 | 0,28 |
| wrist circ | Females | 13 | 69 | 39,59 | 98,73 | 12,0710 | 0,28 |
| left forearm circ | Females | 14 | 67 | 38,23 | 95,53 | 12,0256 | 0,27 |
| len_neck_ri_bust_waist | Females | 15 | 69 | 38,41 | 98,93 | 11,4460 | 0,28 |
| chest depth | Females | 16 | 66 | 36,79 | 94,97 | 11,4098 | 0,28 |
| buttock circ | Females | 17 | 67 | 37,11 | 96,37 | 11,3049 | 0,28 |
| belly circ | Females | 18 | 66 | 36,45 | 95,37 | 11,1763 | 0,28 |
| high hip circ | Females | 19 | 66 | 36,45 | 95,47 | 11,1588 | 0,28 |
| right elbow circ | Females | 20 | 66 | 36,62 | 96,22 | 11,0958 | 0,28 |
| circ_max_belly | Females | 21 | 66 | 36,39 | 95,75 | 11,0797 | 0,28 |
| circ_underbust_horiz | Females | 22 | 66 | 36,27 | 96,29 | 10,9247 | 0,27 |
| circ_middle_hip | Females | 23 | 66 | 36,26 | 96,52 | 10,8798 | 0,27 |
| calf_ankle_ratio_r | Females | 24 | 64 | 34,41 | 93,27 | 10,2878 | 0,27 |
| circ_waist | Females | 25 | 64 | 34,10 | 94,30 | 10,2258 | 0,27 |
| chest_breadth | Females | 26 | 61 | 32,11 | 89,17 | 10,1421 | 0,27 |
| circ_wrist_left | Females | 27 | 68 | 36,09 | 100,57 | 10,1318 | 0,28 |
| circ_wrist_right | Females | 28 | 68 | 36,01 | 100,47 | 10,1194 | 0,29 |
| circ_ankle_right | Females | 29 | 65 | 34,35 | 96,43 | 10,0692 | 0,27 |
| hip_breadth | Females | 30 | 61 | 31,66 | 89,50 | 9,9693 | 0,27 |
| circ_elbow_left | Females | 31 | 60 | 30,86 | 88,48 | 9,7739 | 0,27 |
| circ_high_waist | Females | 32 | 62 | 31,97 | 92,67 | 9,6353 | 0,27 |
| circ_upperarm_right | Females | 33 | 59 | 30,15 | 88,41 | 9,4851 | 0,27 |
| circ_knee_right | Females | 34 | 59 | 29,23 | 89,55 | 8,9623 | 0,27 |
| circ_knee_left | Females | 35 | 60 | 29,46 | 90,34 | 8,9547 | 0,27 |
| len_cross_shoul_neck | Females | 36 | 56 | 27,47 | 84,39 | 8,9327 | 0,27 |
| len_cross_shoul | Females | 37 | 55 | 27,11 | 83,43 | 8,9124 | 0,27 |
| circ_ankle_left | Females | 38 | 61 | 29,71 | 92,01 | 8,8462 | 0,27 |
| thigh_knee_ratio_r | Females | 39 | 63 | 30,41 | 96,15 | 8,3567 | 0,28 |
| neck_circumference | Females | 40 | 56 | 26,21 | 85,13 | 8,2124 | 0,27 |
| buttock_knee_len | Females | 41 | 66 | 29,47 | 102,73 | 7,7287 | 0,27 |
| butt_abdomen_depth | Females | 42 | 55 | 24,56 | 86,36 | 7,6556 | 0,27 |
| len_crotch_back | Females | 43 | 53 | 22,61 | 82,61 | 7,4466 | 0,27 |
| thigh_knee_ratio_l | Females | 44 | 58 | 24,61 | 91,57 | 7,3650 | 0,27 |
| circ_total_torso | Females | 45 | 54 | 21,52 | 86,30 | 6,7426 | 0,26 |
| knee_height | Females | 46 | 69 | 24,67 | 114,05 | 6,0033 | 0,26 |
| len_crotch | Females | 47 | 45 | 13,83 | 75,41 | 5,3640 | 0,26 |
| len_waist_thigh_right | Females | 48 | -39 | -65,40 | -11,78 | 5,3048 | 0,26 |
| abdominal_depth | Females | 49 | 45 | 13,59 | 76,01 | 5,2802 | 0,26 |
| len_torso_width_waist | Females | 50 | 42 | 11,45 | 73,09 | 4,9041 | 0,26 |
| len_upperarm_diam_rght | Females | 51 | 40 | 10,18 | 69,20 | 4,7513 | 0,26 |
| shoulder_elbow_len | Females | 52 | -50 | -88,09 | -11,23 | 4,4562 | 0,26 |
| elbow_wrist_len | Females | 53 | 45 | 8,71 | 82,13 | 4,1578 | 0,26 |
| len_upperarm_diam_left | Females | 54 | 37 | 7,02 | 67,70 | 4,1267 | 0,26 |
| Forearm-fingertip_len | Females | 55 | 40 | 0,86 | 79,74 | 3,0862 | 0,26 |
| len_crotch_front | Females | 56 | 31 | 0,25 | 62,39 | 3,0215 | 0,25 |
| len_forehead | Females | 57 | -28 | -55,80 | 0,62 | 2,8865 | 0,26 |
| len_arm_right | Females | 58 | -38 | -79,39 | 3,29 | 2,6332 | 0,25 |
| circ_head | Females | 59 | 25 | -2,40 | 53,22 | 2,6046 | 0,26 |
| len_upperarm_right | Females | 60 | -34 | -72,32 | 3,52 | 2,5776 | 0,25 |
| upper_elbow_ratio_l | Females | 61 | 24 | -4,83 | 53,33 | 1,9942 | 0,25 |
| len_leg_ankle_left_med | Females | 62 | -43 | -99,35 | 14,13 | 1,9547 | 0,25 |
| len_arm_c7_right | Females | 63 | 31 | -13,49 | 75,15 | 1,7525 | 0,25 |
| len_arm_c7_left | Females | 64 | 24 | -18,26 | 66,92 | 1,3341 | 0,25 |
| len_upperarm_left | Females | 65 | -21 | -58,65 | 16,41 | 1,3076 | 0,25 |
| len_waist_buttock_rght | Females | 66 | -20 | -56,00 | 15,84 | 1,2951 | 0,25 |
| len_waist_buttock_left | Females | 67 | -20 | -55,70 | 15,96 | 1,2816 | 0,25 |
| len_leg_right_lat | Females | 68 | 28 | -27,72 | 83,30 | 1,1179 | 0,25 |
| elbow_grip_len | Females | 69 | 18 | -18,26 | 54,02 | 1,1006 | 0,25 |
| len_leg_left_lat | Females | 70 | 27 | -28,56 | 83,54 | 1,0880 | 0,25 |
| len_leg_ankle_rght_lat | Females | 71 | 24 | -28,26 | 76,96 | 1,0087 | 0,25 |
| len_leg_ankle_left_lat | Females | 72 | 24 | -28,99 | 77,29 | 0,9849 | 0,25 |
| upper_elbow_ratio_r | Females | 73 | 11 | -17,48 | 40,08 | 0,8729 | 0,25 |
| len_forearm_right | Females | 74 | -12 | -44,64 | 20,16 | 0,7780 | 0,25 |
| sitting_height | Females | 75 | 19 | -33,06 | 71,80 | 0,7566 | 0,26 |
| len_waist_thigh_left | Females | 76 | -10 | -37,85 | 17,49 | 0,7529 | 0,25 |
| len_waist_high_hip | Females | 77 | -9 | -41,95 | 23,83 | 0,5286 | 0,24 |
| len_upper_torso_left | Females | 78 | 8 | -22,34 | 38,74 | 0,5124 | 0,25 |
| crotch_height | Females | 79 | 14 | -48,45 | 77,41 | 0,4274 | 0,25 |
| len_arm_left | Females | 80 | -9 | -49,10 | 31,96 | 0,3876 | 0,24 |
| len_upper_torso_right | Females | 81 | 6 | -23,56 | 36,04 | 0,3830 | 0,25 |
| len_leg_ankle_rght_med | Females | 82 | 5 | -27,16 | 36,50 | 0,2565 | 0,25 |
| deg_upper_torso | Females | 83 | 4 | -23,44 | 30,88 | 0,2380 | 0,25 |
| len_c7_waist | Females | 84 | 4 | -28,33 | 37,21 | 0,2347 | 0,25 |
| len_forearm_left | Females | 85 | 3 | -30,33 | 37,25 | 0,1733 | 0,24 |
| len_waist_buttock | Females | 86 | -1 | -38,53 | 36,53 | 0,0423 | 0,25 |
| stature=height | Females | 87 | -1 | -222,05 | 220,27 | 0,0063 | 0,26 |
| left thigh knee ratio | Males | 1 | 140 | 77,84 | 201,82 | 12,3225 | 0,39 |
| right thigh knee ratio | Males | 2 | 126 | 65,90 | 186,16 | 10,4024 | 0,38 |
| left upperarm circ | Males | 3 | 93 | 41,42 | 144,58 | 7,7119 | 0,38 |
| right thigh circ | Males | 4 | 93 | 39,89 | 145,89 | 7,3519 | 0,38 |
| left thigh circ | Males | 5 | 97 | 41,36 | 152,26 | 7,3051 | 0,38 |
| right upperarm circ | Males | 6 | 89 | 37,14 | 141,46 | 7,0676 | 0,37 |
| right calf circ | Males | 7 | 75 | 25,00 | 124,68 | 5,6851 | 0,37 |
| left forearm circ | Males | 8 | 76 | 24,82 | 127,74 | 5,5648 | 0,37 |
| Forearm-fingertip len | Males | 9 | 102 | 30,99 | 173,99 | 5,2675 | 0,37 |
| right forearm circ | Males | 10 | 72 | 21,40 | 123,22 | 5,1902 | 0,37 |
| left calf circ | Males | 11 | 64 | 14,24 | 113,24 | 4,4298 | 0,37 |
| left upper elbow ratio | Males | 12 | 60 | 13,19 | 106,83 | 4,0749 | 0,37 |
| thigh thickness | Males | 13 | 62 | 11,16 | 113,72 | 4,0527 | 0,37 |
| left upperarm diam | Males | 14 | 60 | 8,52 | 110,48 | 3,9738 | 0,37 |
| right upper elbow ratio | Males | 15 | 61 | 8,52 | 112,84 | 3,7914 | 0,36 |
| len waist buttock | Males | 16 | 73 | 8,03 | 138,03 | 3,5720 | 0,37 |
| right elbow circ | Males | 17 | 53 | 5,59 | 101,27 | 3,5703 | 0,37 |
| sitting_height | Males | 18 | 89 | 8,26 | 170,26 | 3,5377 | 0,37 |
| left elbow circ | Males | 19 | 54 | 4,76 | 102,34 | 3,4652 | 0,37 |
| right upperarm diam | Males | 20 | 53 | 3,63 | 102,49 | 3,4437 | 0,37 |
| stature=height | Males | 21 | 364 | 21,04 | 706,70 | 3,3279 | 0,37 |
| circ_wrist_left | Males | 22 | 56 | 2,85 | 108,85 | 3,2700 | 0,37 |
| len_forearm_right | Males | 23 | 63 | 2,56 | 123,54 | 3,2330 | 0,36 |
| calf_ankle_ratio_r | Males | 24 | 56 | 1,98 | 110,00 | 3,1803 | 0,37 |
| circ_middle_hip | Males | 25 | -50 | -97,95 | -1,67 | 3,1438 | 0,36 |
| len_waist_buttock_left | Males | 26 | 65 | 0,41 | 129,93 | 3,0138 | 0,36 |
| bideltoid_breadth | Males | 27 | 54 | -0,19 | 108,01 | 2,9689 | 0,36 |
| len_c7_waist | Males | 28 | -60 | -122,31 | 1,33 | 2,8877 | 0,36 |
| len_forearm_left | Males | 29 | 49 | -1,29 | 100,05 | 2,8697 | 0,37 |
| abdominal_depth | Males | 30 | -46 | -93,46 | 1,42 | 2,8504 | 0,36 |
| len_waist_buttock_rght | Males | 31 | 63 | -2,44 | 127,50 | 2,8168 | 0,36 |
| circ_ankle_left | Males | 32 | 48 | -1,98 | 98,66 | 2,8083 | 0,37 |
| circ_wrist_right | Males | 33 | 50 | -2,17 | 101,41 | 2,7966 | 0,37 |
| len_arm_right | Males | 34 | 61 | -3,46 | 126,12 | 2,7468 | 0,36 |
| len_arm_left | Males | 35 | 59 | -4,25 | 122,01 | 2,6858 | 0,37 |
| knee_height | Males | 36 | -72 | -151,68 | 7,46 | 2,5733 | 0,36 |
| circ_ankle_right | Males | 37 | 44 | -4,85 | 93,53 | 2,5526 | 0,37 |
| circ_high_hip | Males | 38 | -43 | -90,68 | 4,98 | 2,5295 | 0,37 |
| len_waist_high_hip | Males | 39 | 52 | -6,21 | 109,79 | 2,5175 | 0,36 |
| circ_wrist | Males | 40 | 43 | -7,18 | 92,28 | 2,3625 | 0,36 |
| len_torso_width_waist | Males | 41 | -41 | -89,54 | 7,42 | 2,3278 | 0,36 |
| butt_abdomen_depth | Males | 42 | -40 | -88,76 | 8,40 | 2,2477 | 0,36 |
| circ_belly | Males | 43 | -39 | -86,68 | 8,86 | 2,1982 | 0,37 |
| circ_max_belly | Males | 44 | -39 | -86,46 | 9,06 | 2,1815 | 0,37 |
| len_arm_c7_left | Males | 45 | 57 | -15,64 | 129,82 | 2,1382 | 0,36 |
| elbow_grip_len | Males | 46 | 49 | -16,06 | 114,24 | 2,0831 | 0,36 |
| calf_ankle_ratio_l | Males | 47 | 40 | -15,30 | 95,64 | 1,9639 | 0,37 |
| len_arm_c7_right | Males | 48 | 51 | -22,77 | 124,25 | 1,7332 | 0,36 |
| len_neck_ri_bust_waist | Males | 49 | 42 | -20,90 | 104,08 | 1,6468 | 0,37 |
| len_forehead | Males | 50 | -28 | -72,97 | 17,27 | 1,4829 | 0,37 |
| circ_waist | Males | 51 | -30 | -77,42 | 18,40 | 1,4790 | 0,36 |
| len_upper_torso_right | Males | 52 | -30 | -79,32 | 18,92 | 1,4751 | 0,36 |
| len_cross_shoul | Males | 53 | -29 | -77,00 | 19,18 | 1,4301 | 0,36 |
| body_depth | Males | 54 | -28 | -76,24 | 19,54 | 1,4001 | 0,36 |
| len_waist_thigh_left | Males | 55 | -26 | -72,66 | 20,10 | 1,3195 | 0,36 |
| len_leg_ankle_rght_med | Males | 56 | -35 | -100,31 | 29,33 | 1,2597 | 0,36 |
| buttock_knee_len | Males | 57 | -39 | -109,92 | 32,54 | 1,2462 | 0,36 |
| len_crotch_front | Males | 58 | -25 | -73,43 | 23,05 | 1,1820 | 0,36 |
| chest_depth | Males | 59 | 25 | -23,98 | 73,38 | 1,1381 | 0,36 |
| len_cross_shoul_neck | Males | 60 | -24 | -71,32 | 23,88 | 1,1110 | 0,36 |
| circ_head | Males | 61 | 24 | -25,76 | 73,14 | 1,0546 | 0,37 |
| circ_high_waist | Males | 62 | -23 | -70,43 | 24,85 | 1,0530 | 0,36 |
| hip_breadth | Males | 63 | -24 | -74,32 | 26,52 | 1,0403 | 0,36 |
| circ_knee_right | Males | 64 | 22 | -26,00 | 70,38 | 1,0021 | 0,36 |
| len_leg_right_lat | Males | 65 | 38 | -47,37 | 122,93 | 0,9549 | 0,36 |
| chest_breadth | Males | 66 | 21 | -27,06 | 68,94 | 0,9341 | 0,36 |
| len_leg_ankle_rght_lat | Males | 67 | 35 | -45,94 | 115,74 | 0,9216 | 0,36 |
| deg_upper_torso | Males | 68 | 19 | -25,72 | 62,94 | 0,8889 | 0,36 |
| crotch_height | Males | 69 | 35 | -49,08 | 119,10 | 0,8799 | 0,36 |
| len_leg_left_lat | Males | 70 | 35 | -51,42 | 120,58 | 0,8415 | 0,36 |
| len_upper_torso_left | Males | 71 | 18 | -29,34 | 65,76 | 0,7911 | 0,36 |
| len_leg_ankle_left_lat | Males | 72 | 31 | -50,62 | 112,72 | 0,7840 | 0,36 |
| len_crotch | Males | 73 | -16 | -65,42 | 33,28 | 0,6468 | 0,36 |
| len_waist_thigh_right | Males | 74 | 15 | -31,27 | 61,01 | 0,6392 | 0,36 |
| circ_knee_left | Males | 75 | 15 | -33,42 | 63,04 | 0,6021 | 0,36 |
| len_upperarm_right | Males | 76 | 17 | -44,72 | 78,24 | 0,5221 | 0,36 |
| shoulder_elbow_len | Males | 77 | -14 | -75,38 | 46,56 | 0,4410 | 0,36 |
| circ_bust | Males | 78 | 11 | -36,02 | 58,28 | 0,4401 | 0,36 |
| circ_underbust_horiz | Males | 79 | 9 | -37,14 | 55,96 | 0,3679 | 0,36 |
| circ_buttock | Males | 80 | -10 | -58,95 | 39,25 | 0,3646 | 0,36 |
| len_upperarm_left | Males | 81 | 11 | -50,86 | 72,26 | 0,3099 | 0,36 |
| circ_total_torso | Males | 82 | -10 | -70,36 | 50,60 | 0,2890 | 0,37 |
| len_leg_ankle_left_med | Males | 83 | 8 | -71,35 | 87,35 | 0,1704 | 0,36 |
| elbow_wrist_len | Males | 84 | 5 | -61,31 | 71,91 | 0,1322 | 0,36 |
| len_crotch_back | Males | 85 | -2 | -51,04 | 48,00 | 0,0492 | 0,36 |
| circ_hip | Males | 86 | 0 | -48,72 | 49,24 | 0,0083 | 0,36 |
| neck_circumference | Males | 87 | 0 | -48,58 | 49,08 | 0,0081 | 0,36 |

1. **Supplementary table 3: Correlation coefficients between the anthropometric markers**

Due to its size this table can be found in the Excel file “Tables”.
